# Demographic influences on Lithuanian physicians’ attitudes toward medical assistance in dying: a cross-sectional study

**DOI:** 10.3389/fpsyt.2024.1507790

**Published:** 2025-01-07

**Authors:** Benedikt Bachmetjev, Artur Airapetian, Marija Jakubauskienė, Rolandas Zablockis, Asta Čekanauskaitė

**Affiliations:** ^1^ Faculty of Medicine, Vilnius University, Vilnius, Lithuania; ^2^ Public Health Department, Faculty of Medicine, Institute of Health Sciences, Vilnius University, Vilnius, Lithuania; ^3^ Clinic of Chest Diseases, Immunology and Allergology, Faculty of Medicine, Institute of Clinical Medicine, Vilnius University, Vilnius, Lithuania; ^4^ The Centre for Health Ethics, Law and History, Faculty of Medicine, Institute of Health Sciences, Vilnius University, Vilnius, Lithuania

**Keywords:** euthanasia, assisted suicide, end-of-life decisions, medical assistance in dying (MAID), Do-Not-Resuscitate (DNR) orders, living wills

## Abstract

**Background:**

The topic of end-of-life decisions is important due to aging populations and the rising number of terminal illnesses like cancer. As more people experience suffering, the ethical, medical, and legal debates of these decisions become significant to healthcare policy. Understanding medical professionals’ attitudes is critical for shaping responsible practices and legislation surrounding end-of-life care.

**Methods:**

This cross-sectional study explores the attitudes of Lithuanian physicians toward medical assistance in dying (MAID), including euthanasia and assisted suicide (E/PAS), as well as other end-of-life decisions such as Do-Not-Resuscitate (DNR) orders and Living Wills, including decisions involving patients diagnosed with mental illnesses. A survey of 361 physicians in Lithuanian hospitals was conducted between October 2022 and July 2024, using hospital intranets and on-site distribution to guarantee representative sample. The survey included demographic factors such as age, gender, religious beliefs, and professional experience. Statistical analysis was performed using SPSS version 26.0 and R software. Chi-square tests, Fisher’s exact tests, and logistic regression models were made to determine relationships, with significance set at p < 0.05.

**Results:**

The analysis showed that 61.2% of physicians supported assisted suicide for terminally ill patients, while only 19.1% supported it for patients with drug-resistant mental illness. Similarly, 61.5% supported euthanasia for terminal illness. Age, religious beliefs, and professional experience were significant determinants of support, with younger and non-religious physicians more likely to endorse E/PAS. Additionally, 92.2% of respondents supported DNR orders with patient consent, though this dropped to 63.1% without patient consent.

**Conclusions:**

Lithuanian physicians’ attitudes toward E/PAS and other end-of-life decisions are strongly influenced by ethical, religious, and professional considerations. Significantly lower acceptance for psychiatric patients indicates higher sensitivity regarding mental competency and the ethics of E/PAS in such cases. These findings provide important insights for policymakers and healthcare providers in crafting informed and ethical E/PAS guidelines.

## 1 Introduction

### 1.1 Terminology

In an aging society, medical end-of-life decisions have become an essential aspect of healthcare. These decisions encompass ethical and practical considerations, including whether to withhold or withdraw treatments, manage pain and symptoms, and provide the psychological, social, and spiritual support for individuals in their final stages of life. These decisions seek to balance the benefits and risks of care while saving the dignity and autonomy of patients (1).

Active euthanasia, a particularly complex and controversial component of end-of-life care, involves the intentional administration of medications or interventions to directly end a patient’s life to stop the intolerable suffering. This practice raises ethical and legal challenges, testing the boundaries of patient autonomy and the responsibilities of healthcare systems (2). Importantly, euthanasia can only be considered under strict conditions, requiring the explicit and voluntary request of the patient, ensuring their decision is informed and autonomous.

Similarly, Physician-Assisted Suicide provides another option for patients experiencing unbearable suffering. In this process, “the patient self-administers medication that was prescribed by a physician,” providing them control over the timing and circumstances of their death while respecting their autonomy (3). Euthanasia and Physician-Assisted Suicide together make up Medical Assistance in Dying (MAID), which allows healthcare professionals to help patients end their lives by either administering life-ending medication directly or providing it for self-administration, always with the patient’s clear and informed consent (4).

Beyond these practices, end-of-life planning often includes advance directives such as Do Not Resuscitate (DNR) orders and living wills. A DNR order specifies that no CPR or life-support measures should be performed if a patient’s heart stops or they stop breathing, ensuring that emergency measures align with their preferences. Living wills, on the other hand, are legal documents that guide healthcare providers by outlining a patient’s wishes for medical treatments in situations where they can no longer communicate (5). Both tools empower individuals to shape their care, ensuring it reflects their values and goals during critical moments. These practices are subjects of ongoing societal debate and present a significant conflict with the Hippocratic Oath, a historically important cornerstone of medical ethics.

### 1.2 Global trends in end-of-life care

The relevance of this issue is increasing due to demographic changes. In 2020, the number of people aged 60 years and older in the world surpassed the number of children younger than 5 years (6), leading to a rise in age-related diseases. Consequently, there were nearly twenty million new cases of cancer in 2022 (7), and cancer is one of the primary chronic illnesses leading to terminal conditions. Thus, the importance of addressing medical end-of-life decisions continues to grow.

Euthanasia was first legalized in the Netherlands (8) and has since been adopted in other countries, including Spain (9) and Portugal (10), reflecting a growing acceptance of this practice. Alternatively, some countries have opted to legalize assisted suicide, with Switzerland (11) standing out as one of the few that permits this option for non-residents. These practices, however, remain deeply complex and controversial, making them difficult to study and discuss, particularly in regions like post-Soviet countries, where clinical medicine is still heavily influenced by paternalistic traditions and limited research exists on the subject. In addition to euthanasia and assisted suicide, the regulation of Do Not Resuscitate (DNR) orders and Living Wills adds another layer of complexity. The legal frameworks for these advance directives vary widely across jurisdictions. For instance, in some countries, a DNR order can be issued not only in written form but also communicated orally, proving the diversity of approaches to end-of-life decision-making (12).

The aim of this study is to evaluate the attitudes of physicians towards medical end-of-life decisions, as their opinions are crucial given their significant responsibilities in any legal actions. Physicians are responsible for predicting the progression of patients’ diseases, prescribing life-terminating drugs, or even administering them in cases of active euthanasia. Unfortunately, there are no studies on physicians’ opinions about medical end-of-life decisions in Lithuania or any of the Baltic States. Several papers have explored this issue, including a study published in 2024 (13). However, that study focused solely on evaluating the general public’s attitudes toward medical end-of-life decisions, rather than those of healthcare professionals.

## 2 Materials and methods

### 2.1 Study overview

Attitudes toward living wills, Do Not Resuscitate (DNR) orders, assisted suicide, active euthanasia for patients with somatic disease as well as for those with a mental disorder were evaluated. The study examined how various socio-demographic factors (age, gender, etc.) influence these practices by utilizing statistical analyses, including logistic regression models.

### 2.2 Data collection

An open cohort approach was applied for data collection during the period from October 19, 2022, to July 12, 2024. The targeted representative sample size was 360, which was determined using GPower software, and the final study sample comprised 361 respondents, representing structured demographics including residence, gender, and age groups. The survey was anonymous, and the participants were additionally informed that their personal data would remain confidential.

### 2.3 Selection criteria

1. Language Proficiency: Fluency in Lithuanian was necessary, as the survey was administered in Lithuanian.
2. Residency: The study was limited to individuals living in Lithuania.
3. Professional Background: Only physicians who had graduated from their residency were considered eligible.
4. Workplace: Only physicians employed in hospitals were invited to participate.
5. Participants were included if they acknowledged and agreed to participate by marking a checkbox indicating their consent after being informed about the purpose of the study and its anonymous nature.

We would like to emphasize that the survey was distributed both online via the “Google Forms” platform through hospital intranets and in physical form on-site, ensuring multiple avenues for participation.

### 2.4 Consent and anonymity

The survey was designed to maintain anonymity. Participants were required to express their consent by checking a box before proceeding, ensuring they understood that their responses would be used anonymously for analysis and publication. This consent process upheld respondent anonymity while fully informing them about their participation in the study.

### 2.5 Survey scenarios

The questionnaire included several clinical cases and nine demographic questions. Six hypothetical medical scenarios were designed using scientific literature and the clinical practices of consulting physicians to explore attitudes toward MAID across various situations. Clinical scenarios were reviewed and validated by the expert group, which included a medical ethicist, chemotherapist, a radiotherapist and a critical care specialist which proved them to be relevant and accurate?

The scenarios were as follows:

#### 2.5.1 Assisted suicide for patient with somatic illness

A case of an advanced bone cancer in a young patient. A 25-year-old patient diagnosed with malignant osteosarcoma, post-surgery and chemotherapy, experiences disease progression with multiple metastases in the lungs and other organs. The patient suffers from constant shortness of breath and pain. The prognosis, confirmed by three independent physicians, is poor, with an expected lifespan of up to six months, and only symptomatic treatment available. The respondents were asked if they supported granting patients the right to terminate their own lives with a physician’s prescription for life-ending drugs.

#### 2.5.2 Active euthanasia for patient with somatic illness

A case of an advanced lung cancer. A 42-year-old patient with small cell lung cancer is hospitalized due to pain in the right side. Examination reveals tumor formations in lymph nodes, bones, and liver (metastases confirmed by a pathologist). As the disease progresses, the patient experiences impaired breathing and swallowing, pain, and inability to walk. Three independent specialists have determined the prognosis to be poor, with a life expectancy of three months and only symptomatic treatment available. The respondents were asked if they supported granting patients the right to terminate their lives upon request through a physician’s injection of life-ending drugs due to a terminal illness.

#### 2.5.3 Do-Not-Resuscitate with a consent of a patient

A 62-year-old patient diagnosed with small cell lung cancer six months ago has undergone an unsuccessful chemotherapy course and is offered only symptomatic treatment, with a life expectancy of about four months. Metastases have been found in other organs. The respondents were asked if they supported granting patients the right to complete a Do-Not-Resuscitate (DNR) order and not to be resuscitated due to a terminal illness.

#### 2.5.4 Do-Not-Resuscitate without a consent of a patient

A 62-year-old patient with small cell lung cancer diagnosed six months ago has undergone unsuccessful chemotherapy and is offered only symptomatic treatment, with a life expectancy of about four months. Metastases are found in other organs. The patient arrives at the hospital with impaired consciousness, unable to walk independently, and not urinating. The respondents were asked if they supported the right of physicians to not resuscitate a patient without the patient’s consent.

#### 2.5.5 Living Will order

The respondents were asked if they believed a mentally and physically healthy individual should have the right to write an advance directive, stating that in the event of severe cognitive and health deterioration with acute, irreversible changes, certain life-sustaining treatments (such as artificial ventilation, dialysis, use of antibiotics, etc.) should not be applied? (commonly known as a “Living Will”).

#### 2.5.6 Assisted suicide for patient with chronic mental illness

A 28-year-old patient has suffered from psychosis and treatment-resistant depression since the age of 12, experiencing constant psychological suffering and multiple suicide attempts. The voices the patient hears continuously urge self-harm. Despite long-term psychotherapy and medication, the patient’s condition has not improved. Over two years, the patient was evaluated by a panel of three independent physicians, who concluded that all possible treatment options had been exhausted. The respondents were asked if they supported granting patients the right to terminate their own lives with a physician’s prescription for life-ending drugs due to a drug-resistant mental illness.

### 2.6 Demographic information

The questionnaire also included demographic information such as age, gender, years of experience in caring for people with terminal condition, overall professional experience, specialty, experience in treating terminally ill patients, place of residence, workplace location, and religious beliefs. These socio-demographic questions were chosen based on precedents set by similar studies in this field. This approach ensured that the data collected was relevant and comparable to existing research, providing a more comprehensive understanding of the subject matter.

### 2.7 Validation and analysis

Before distribution, the questionnaire was reviewed for face validity to ensure the questions were appropriate and relevant to the study’s goals. The questions, tailored to the Lithuanian context, were clear and concise. Each item was assessed individually and did not contribute to a total score, making this validation sufficient. Participants had no time constraints for the completion of the survey.

Descriptive and analytical statistical methods were employed to analyze the data, with a 95% confidence interval used for prevalence values. Logistic regression models were created using SPSS version 26.0 (IBM Corp, Armonk, NY, USA) and R. For more detailed data analysis, Fisher’s exact test and Pearson’s chi-squared test were utilized, with statistical significance defined as p < 0.05.

### 2.8 Ethical considerations

According to Lithuanian law, this anonymous survey did not require ethical approval as it did not meet the legal criteria for a biomedical study. This was possible on two key conditions:

1. Anonymity: The survey was entirely anonymous. No names, surnames, IP addresses, or any other identifying information were collected. Participants filled out the questionnaire independently, and there was no way for us to trace their responses back to them, ensuring complete confidentiality.
2. Informed Participation: All participants were fully informed about the purpose and nature of the study, including the sensitive nature of the topic. A detailed explanation was provided at the beginning of the survey, allowing participants to make an informed decision about whether to proceed. By completing the survey, they indicated their consent to participate.

Every effort was made to ensure that participants experienced no psychological discomfort through clear and comprehensive information about the purpose and nature of the study. Simultaneously, strict measures were implemented to guarantee anonymity, allowing participants to share their views confidentially and without concern for identification.

## 3 Results

### 3.1 Demographic characteristics of the participants

Analyzing the data in **Table 1A**, we see the demographic characteristics of the survey participants:

**Table 1A:** The demographical features of the respondents.

Demographic Variables		Number (Percent) of Respondents
Gender	Male	128 (35.5)
	Female	233 (64.5)
Age of respondents	< 35 years	120 (33.2)
	35-45 years	58 (16.1)
	46-55 years	90 (24.9)
	> 55 years	93 (25.8)
Place of residence	Rural	20 (5.5)
	Urban	341 (94.5)
Residence of the workplace	Rural	20 (5.5)
	Urban	341 (94.5)
Religion	Religious	90 (24.9)
	Non-religious	271 (75.1)
Number of years of professional experience	< 10 years	136 (37.7)
	10-20 years	80 (22.1)
	21-30 years	71 (19.7)
	> 30 years	74 (20.5)
Experience in caring of patients with a terminal condition	No experience	166 (46.0)
	< 1 year	113 (31.3)
	1 year to 5 years	45 (12.4)
	> 5 years	37 10.3)
Presence of experience in treating terminally ill patients	Present	264 (73.1)
	Not present	87 (24.1)
	Can’t answer	10 (2.8)
Specialty	Surgical	70 (19.4)
	Therapeutical	291 (80.6)

**Table 1B:** Age characteristics of the participants.

Group	Mean	St. deviation	0%	25%	50%	75%	100%
Male	44.3	13.7	24	31	44	56	70
Female	45.3	13.2	25	32	46	56	77
Overall	45.0	13.3	24	32	46	56	77

#### 3.1.1 Qualitative characteristics

Gender: Most respondents were female, making up 64.5% (n=233) of the sample, while males accounted for 35.5% (n=128).

Residency: The majority of respondents, 94.5% (n=341), both lived and worked in urban areas, while only 5.5% (n=20) resided and worked in rural locations.

Religion: Regarding religious affiliation, 75.1% (n=271) of respondents identified as non-religious, while 24.9% (n=90) identified as religious.

Specialty: Regarding the specialties of the respondents, 80.6% (n=291) worked in therapeutic fields, while 19.4% (n=70) were in surgical fields. The difference between surgical and therapeutical specialties in the context of this exact study was made as follows:

Therapeutic fields were considered: Family Medicine, Neurology, Pulmonology, Infectious Diseases, Gastroenterology, Radiation Oncology, Anesthesiology and Reanimatology, Emergency Medicine, Pediatrics, Geriatrics, Internal Medicine, Pediatrics and Pediatric Neurology, Radiology, Psychiatry, Pediatrics and Neonatology, Pediatrics and Pediatric Rheumatology, Dermatology and Venereology, Pediatrics and Pediatric Nephrology, Child and Adolescent Psychiatry, Rheumatology, Hematology, Pediatrics and Pediatric Gastroenterology, Allergy and Clinical Immunology, Physical Medicine and Rehabilitation, Genetics, Chemotherapeutic Oncology, Endocrinology, Nephrology, Pediatrics and Pediatric Cardiology, Laboratory Medicine, Pediatrics and Pediatric Intensive Care, Forensic Medicine, Laboratory Medicine, Dietology.

Surgical fields were considered: Surgery, Maxillofacial Surgery, Urology, Pediatric Surgery, Orthopedics and Traumatology, Obstetrics and Gynecology, Vascular Surgery, Plastic and Reconstructive Surgery, Abdominal Surgery, Thoracic Surgery.

#### 3.1.2 Quantitative Characteristics

Age: The age distribution of the respondents was as follows: 33.2% (n=120) were under 35 years old, 16.1% (n=58) were aged between 35 and 45, 24.9% (n=90) were between 46 and 55 years old, and 25.8% (n=93) were over 55 years old. As seen from a **Table 1B**, the average age of male participants was 44.3 years (± 13.7), while female participants had a slightly higher average age of 45.3 years (± 13.2). The overall mean age was 45.0 years (± 13.3). Percentiles indicate that 50% of participants were aged 44-46 years, with the 25th and 75th percentiles ranging from 31-56 years for males and 32-56 years for females.

Caring experience: In terms of experience in caring for terminally ill patients, 46.0% (n=166) had no experience, 31.3% (n=113) had less than one year of experience, 12.4% (n=45) had one to five years of experience, and 10.3% (n=37) had more than five years of experience. Additionally, 73.1% (n=264) had experience treating terminally ill patients, 24.1% (n=87) did not, and 2.8% (n=10) were unsure.

Professional experience: The number of years of professional experience among respondents varied: 37.7% (n=136) had less than 10 years of experience, 22.1% (n=80) had 10 to 20 years, 19.7% (n=71) had 21 to 30 years, and 20.5% (n=74) had more than 30 years of such experience.

### 3.2 Acceptance of end-of-life decisions across different clinical scenarios

**Table 2** illustrates physicians’ acceptance of various end-of-life decisions in different clinical scenarios. In the scenario describing assisted suicide for terminally ill patients with somatic disorder, 61.2% (n=221) of respondents supported the decision, while 21.1% (n=76) did not, and 17.7% (n=64) were undecided. However, the acceptance of assisted suicide for patients with drug-resistant mental illness was notably lower, with only 19.1% (n=69) in support, 53.2% (n=192) opposed, and 27.7% (n=100) unable to decide. Euthanasia in terminal illness, on the other hand, was accepted by 61.5% (n=222) of respondents, with 24.1% (n=87) disapproving and 14.4% (n=52) uncertain. This level of acceptance is comparable to that of assisted suicide in cases of somatic illness.

**Table 2:** Acceptance of end-of-life decisions in various clinical scenarios.

Clinical case	Number of Respondents		
	"Yes"	"No"	"Can't decide"
A case describing a process of assisted suicide in terminal illness	221 (61.2%)	76 (21.1%)	64 (17.7%)
A case describing a process of euthanasia in terminal illness	222 (61.5%)	87 (24.1%)	52 (14.4%)
A case of Do-Not-Resuscitate Order (DNR) in terminal illness	333 (92.2%)	14 (3.9%)	14 (3.9%)
A case of DNR in terminal illness without a consent of the patient	228 (63.1%)	88 (24.4%)	45 (12.5%)
A case of Living Will order for a Healthy Individual	254 (70.4%)	66 (18.2%)	41 (11.4%)
A case of assisted suicide in drug-resistant mental illness	69 (19.1%)	192 (53.2%)	100 (27.7%)

In the case of Do-Not-Resuscitate (DNR) orders, 92.2% (n=333) of physicians approved when the patient had given consent, while only 3.9% (n=14) disapproved and 3.9% (n=14) were undecided. Without the patient’s consent, approval dropped to 63.1% (n=228), with 24.4% (n=88) disapproving and 12.5% (n=45) uncertain. For Living Will orders in healthy individuals, 70.4% (n=254) were in favor, 18.2% (n=66) were against, and 11.4% (n=41) were unsure.

### 3.3 Factors Influencing physicians’ attitudes towards assisted suicide (due to mental or somatic illness) and euthanasia

The datasets in **Tables 3**, **4** compare the attitudes of Lithuanian physicians toward assisted suicide for patients with a somatic (terminal) illness as well as with drug-resistant mental illness.

**Table 3:** The distribution of answers in the clinical case regarding assisted suicide according to different characteristics of respondents.

Demographic Variables		Number of Respondents			Significance		
		"Yes"	"No"	"Can't decide"	X²	df	*P*-value
**Gender**	Male	88 (68.8%)	24 (18.8%)	16 (12.5%)	5.395	2	0.067
	Female	133 (57.1%)	52 (22.3%)	48 (20.6%)			
**Age of respondents**	< 35 years	99 (82.5%)	9 (7.5%)	12 (10.0%)	53.814	6	**0.000**
	35-45 years	39 (67.2%)	5 (8.6%)	14 (24.1%)			
	46-55 years	39 (43.3%)	29 (32.2%)	22 (24.4%)			
	> 55 years	44 (47.3%)	33 (35.5%)	16 (17.2%)			
**Religion**	Religious	149 (55.0%)	69 (25.5%)	69 (13.0%)	18.994	2	**0.000**
	Non-religious	72 (80.0%)	7 (7.8%)	592 (11.3%)			
**Place of residency**	Urban	207 (60.7%)	74 (21.7%)	60 (17.6%)	1.557	2	0.459
	Rural	14 (70.0%)	2 (10.0%)	4 (20.0%)			
**Place of workplace**	Urban	216 (60.7%)	76 (21.3%)	64 (18.0%)	3.212	2	0.201
	Rural	5 (100.0%)	0 (0.0%)	0 (0.0%)			
**Specialty**	Therapeutical	172 (59.1%)	65 (22.3%)	54 (18.6%)	2.850	2	0.241
	Surgical	49 (70.0%)	11 (15.7%)	10 (14.3%)			
**Professional** **experience**	< 10 years	109 (80.1%)	14 (10.3%)	13 (9.6%)	55.948	6	**0.000**
	11-20 years	41 (57.7%)	7 (9.9%)	23 (32.4%)			
	21-30 years	74 (100.0%)	24 (32.4%)	14 (18.9%)			
	> 30 years	35 (43.8%)	31 (38.8%)	14 (17.5%)			
**Experience in caring of patients with a terminal condition**	No experience	117 (70.5%)	24 (14.5%)	25 (15.1%)	25.416	6	**0.000**
	< 1 year	70 (61.9%)	25 (22.1%)	18 (15.9%)			
	1 year to 5 years	23 (51.1%)	14 (31.1%)	8 (17.8%)			
	> 5 years	11 (29.7%)	13 (35.1%)	13 (35.1%)			
**Experience in treating terminally ill**	Present	161 (61.0%)	61 (23.1%)	42 (15.9%)	3.811	2	0.149
	Non-present	60 (61.9%)	15 (15.5%)	22 (22.7%)			

Significant values are shown in bold.

**Table 4:** The distribution of answers in the clinical case regarding assisted suicide due to mental illness according to different characteristics of respondents.

Demographic Variables		Number of Respondents			Significance		
		"Yes"	"No"	"Can't decide"	X²	df	*P*-value
**Gender**	Male	24 (18.8%)	71 (55.5%)	33 (25.8%)	0.472	2	0.790
	Female	45 (19.3%)	121 (51.9%)	67 (28.8%)			
**Age of** **respondents**	< 35 years	32 (26.7%)	48 (40.0%)	40 (33.3%)	18.352	6	**0.005**
	35-45 years	12 (20.7%)	30 (51.7%)	16 (27.6%)			
	46-55 years	9 (10.0%)	55 (61.1%)	26 (28.9%)			
	> 55 years	16 (17.2%)	59 (63.4%)	18 (19.4%)			
**Religion**	Religious	41 (15.1%)	153 (56.5%)	69 (13.0%)	11.416	2	**0.003**
	Non-religious	28 (31.1%)	39 (43.3%)	592 (11.3%)			
**Place of** **residency**	Urban	63 (18.5%)	184 (54.0%)	94 (27.6%)	2.045	2	0.360
	Rural	6 (30.0%)	8 (40.0%)	6 (30.0%)			
**Place of** **workplace**	Urban	67 (18.8%)	190 (53.4%)	99 (27.8%)	1.432	2	0.489
	Rural	2 (40.0%)	2 (40.0%)	1 (20.0%)			
**Specialty**	Therapeutical	51 (17.5%)	161 (55.3%)	79 (27.1%)	3.438	2	0.179
	Surgical	18 (25.7%)	31 (44.3%)	21 (30.0%)			
**Professional** **experience**	< 10 years	36 (26.5%)	58 (42.6%)	42 (30.9%)	15.328	6	**0.018**
	11-20 years	13 (18.3%)	37 (52.1%)	21 (29.6%)			
	21-30 years	74 (100.0%)	45 (60.8%)	21 (28.4%)			
	> 30 years	12 (15.0%)	52 (65.0%)	16 (20.0%)			
**Experience in caring of patients** **with a terminal condition**	No experience	37 (22.3%)	80 (48.2%)	49 (29.5%)	6.705	6	0.349
	< 1 year	19 (16.8%)	62 (54.9%)	32 (28.3%)			
	1 year to 5 years	8 (17.8%)	24 (53.3%)	13 (28.9%)			
	> 5 years	5 (13.5%)	26 (70.3%)	6 (16.2%)			
**Experience in treating terminally ill**	Present	45 (17.0%)	142 (53.8%)	77 (29.2%)	3.028	2	0.220
	Non-present	24 (24.7%)	50 (51.5%)	23 (23.7%)			

Significant values are shown in bold.

The attitudes toward assisted suicide for terminally ill patients (**Table 3**) were influenced by several demographic factors. Gender differences were observed, but they were not statistically significant. Age played a significant role, with the highest support among respondents under the age of 35 (82.5%) and the lowest among those aged 46-55 (43.3%) (p < 0.001). Religiosity was also a significant factor, as 80.0% of non-religious respondents supported assisted suicide compared to 55.0% of religious respondents (p < 0.001). Factors such as place of residence, workplace, and specialty did not show significant associations. Professional experience significantly influenced attitudes, with respondents with less than 10 years of professional experience more likely to support assisted suicide (80.1%) than those with over 30 years of experience (43.8%) (p < 0.001). Additionally, physicians with no experience in caring for terminal patients were more supportive (70.5%) compared to those with over 5 years of experience (29.7%) (p < 0.001).

**Table 4**, which focuses on attitudes toward allowing patients with drug-resistant mental illness to end their lives with a physician’s prescription, shows different trends. Gender was not significantly related to opinions. Age again played a significant role, with the highest support seen among physicians under 35 (26.7%) and the lowest among those aged 46-55 (10.0%) (p = 0.005). Religiosity significantly affected attitudes, with 31.1% of non-religious respondents in support compared to 15.1% of religious respondents (p = 0.003). Factors such as place of residence, workplace, and specialty were not significantly associated with attitudes. Professional experience showed a similar trend, with physicians with less than 10 years of experience more likely to support the decision (26.5%) compared to those with over 30 years of experience (15.0%) (p = 0.018). Experience in caring for terminal patients did not significantly impact attitudes (p = 0.349).

**Table 5** presents the distribution of responses to active euthanasia according to the same demographic variables. In terms of gender, there was no statistically significant difference (p = 0.153). Age was significantly related to the attitudes towards active euthanasia, with the highest support among respondents under 35 years (78.3%; n=94) and the lowest among those aged 46-55 years (46.7%; n=42) (p < 0.001). Religious affiliation also showed a significant relationship, with non-religious respondents showing higher support (75.6%; n=68) compared to religious respondents (56.8%; n=154) (p = 0.004).

**Table 5:** The distribution of answers in the clinical case regarding active euthanasia according to different characteristics of respondents.

Demographic Variables		Number of Respondents			Significance		
		"Yes"	"No"	"Can't decide"	X²	df	*P*-value
**Gender**	Male	87 (68.0%)	27 (21.1%)	14 (10.9%)	3.750	2	0.153
	Female	135 (57.9%)	60 (25.8%)	38 (16.3%)			
**Age of** **respondents**	< 35 years	94 (78.3%)	13 (10.8%)	13 (10.8%)	34.443	6	**0.000**
	35-45 years	38 (65.5%)	14 (24.1%)	6 (10.3%)			
	46-55 years	42 (46.7%)	26 (28.9%)	22 (24.4%)			
	> 55 years	48 (51.6%)	34 (36.6%)	11 (11.8%)			
**Religion**	Religious	154 (56.8%)	76 (28.0%)	69 (13.0%)	11.268	2	**0.004**
	Non-religious	68 (75.6%)	11 (12.2%)	592 (11.3%)			
**Place of** **residency**	Urban	209 (61.3%)	83 (24.3%)	49 (14.4%)	0.195	2	0.907
	Rural	13 (65.0%)	4 (20.0%)	3 (15.0%)			
**Place of** **workplace**	Urban	218 (61.2%)	86 (24.2%)	52 (14.6%)	1.048	2	0.592
	Rural	4 (80.0%)	1 (20.0%)	0 (0.0%)			
**Specialty**	Therapeutical	175 (60.1%)	73 (25.1%)	43 (14.8%)	1.200	2	0.549
	Surgical	47 (67.1%)	14 (20.0%)	9 (12.9%)			
**Professional** **experience**	< 10 years	104 (76.5%)	18 (13.2%)	14 (10.3%)	26.334	6	**0.000**
	11-20 years	42 (59.2%)	16 (22.5%)	13 (18.3%)			
	21-30 years	74 (100.0%)	23 (31.1%)	14 (18.9%)			
	> 30 years	39 (48.8%)	30 (37.5%)	11 (13.8%)			
**Experience in** **caring of patients with a terminal** **condition**	No experience	117 (70.5%)	24 (14.5%)	25 (15.1%)	22.099	6	**0.001**
	< 1 year	68 (60.2%)	32 (28.3%)	13 (11.5%)			
	1 year to 5 years	23 (51.1%)	15 (33.3%)	7 (15.6%)			
	> 5 years	14 (37.8%)	16 (43.2%)	7 (18.9%)			
**Experience in treating terminally ill**	Present	157 (59.5%)	70 (26.5%)	37 (14.0%)	3.138	2	0.208
	Non-present	65 (67.0%)	17 (17.5%)	15 (15.5%)			

Significant values are shown in bold.

The place of residency, workplace, and specialty did not significantly influence attitudes (p = 0.907, p = 0.592, and p = 0.549, respectively). Professional experience was significantly related to the attitudes, with 76.5% (n=104) of respondents with less than 10 years of experience supporting active euthanasia compared to 48.8% (n=39) of those with more than 30 years of experience (p < 0.001). Experience in caring for patients with terminal conditions significantly influenced attitudes, with 70.5% (n=117) of those with no experience supporting active euthanasia compared to 37.8% (n=14) of those with over 5 years of experience (p < 0.001). Experience in treating terminally ill patients did not significantly affect attitudes (p = 0.208).

### 3.4 Factors Influencing physicians’ attitudes towards Living Wills and DNR orders

The dataset shown in **Table 6** represents the attitudes of Lithuanian physicians towards Do-Not-Resuscitate (DNR) orders for lung cancer patients. Gender and age did not significantly affect attitudes (p = 0.732 and p = 0.132, respectively). Religiosity, however, significantly influenced attitudes, with 90.0% (n=244) of religious respondents supporting the DNR order compared to 98.9% (n=89) of non-religious respondents (p = 0.022).

**Table 6:** The distribution of answers in the clinical case regarding DNR order according to different characteristics of respondents.

Demographic Variables		Number of Respondents			Significance		
		"Yes"	"No"	"Can't decide"	X²	df	*P*-value
**Gender**	Male	118 (92.2%)	6 (4.7%)	4 (3.1%)	0.625	2	0.732
	Female	215 (92.3%)	8 (3.4%)	10 (4.3%)			
**Age of** **respondents**	< 35 years	118 (98.3%)	1 (0.8%)	1 (0.8%)	9.833	6	0.132
	35-45 years	52 (89.7%)	3 (5.2%)	3 (5.2%)			
	46-55 years	81 (90.0%)	4 (4.4%)	5 (5.6%)			
	> 55 years	82 (88.2%)	6 (6.5%)	5 (5.4%)			
**Religion**	Religious	244 (90.0%)	13 (4.8%)	69 (13.0%)	7.590	2	**0.022**
	Non-religious	89 (98.9%)	1 (1.1%)	592 (11.3%)			
**Place of** **residency**	Urban	314 (92.1%)	14 (4.1%)	13 (3.8%)	0.907	2	0.635
	Rural	19 (95.0%)	0 (0.0%)	1 (5.0%)			
**Place of** **workplace**	Urban	328 (92.1%)	14 (3.9%)	14 (3.9%)	0.426	2	0.808
	Rural	5 (100.0%)	0 (0.0%)	0 (0.0%)			
**Specialty**	Therapeutical	269 (92.4%)	11 (3.8%)	11 (3.8%)	0.081	2	0.960
	Surgical	64 (91.4%)	3 (4.3%)	3 (4.3%)			
**Professional** **experience**	< 10 years	132 (97.1%)	3 (2.2%)	1 (0.7%)	13.400	6	**0.037**
	11-20 years	64 (90.1%)	1 (1.4%)	6 (8.5%)			
	21-30 years	74 (100.0%)	4 (5.4%)	3 (4.1%)			
	> 30 years	70 (87.5%)	6 (7.5%)	4 (5.0%)			
**Experience in** **caring of patients with a terminal** **condition**	No experience	157 (94.6%)	2 (1.2%)	7 (4.2%)	13.791	6	**0.032**
	< 1 year	107 (94.7%)	5 (4.4%)	1 (0.9%)			
	1 year to 5 years	38 (84.4%)	4 (8.9%)	3 (6.7%)			
	> 5 years	31 (83.8%)	3 (8.1%)	3 (8.1%)			
**Experience in treating terminally ill**	Present	244 (92.4%)	11 (4.2%)	9 (3.4%)	0.772	2	0.680
	Non-present	89 (91.8%)	3 (3.1%)	5 (5.2%)			

Significant values are shown in bold.

The place of residency, workplace, and specialty also did not significantly influence attitudes (p = 0.635, p = 0.808, and p = 0.960, respectively). Professional experience significantly affected attitudes, with 97.1% (n=132) of respondents with less than 10 years of experience supporting the DNR order compared to 87.5% (n=70) among those with more than 30 years of experience (p = 0.037). Experience in caring for patients with terminal conditions also had a significant impact, with 94.6% (n=157) of those with no experience supporting the DNR order compared to 83.8% (n=31) of those with over 5 years of experience (p = 0.032). Experience in treating terminally ill patients did not significantly affect attitudes (p = 0.680).

**Table 7** illustrates the perspectives of Lithuanian physicians on do not resuscitating patients without their consent. A total of 65.6% (n=84) of male respondents and 61.8% (n=144) of female respondents were in favor of not resuscitating without consent. Gender was not a significant factor on opinions (p = 0.728). Age groups also showed no statistically significant influence on approval opinions- the highest support was found among those over 55 years old (67.7%; n=63) and the lowest among those aged 46-55 years (60.0%; n=54) (p = 0.755). Religiosity played a significant role in shaping attitudes, with 58.7% (n=159) of religious respondents supporting the decision compared to 76.7% (n=69) of non-religious respondents. This difference was statistically significant (p = 0.009).

**Table 7:** The distribution of answers in the clinical case regarding DNR order without the consent of a patient according to different characteristics of respondents.

Demographic Variables		Number of Respondents			Significance		
		"Yes"	"No"	"Can't decide"	X²	df	*P*-value
**Gender**	Male	84 (65.6%)	30 (23.4%)	14 (10.9%)	0.634	2	0.728
	Female	144 (61.8%)	58 (24.9%)	31 (13.3%)			
**Age of** **respondents**	< 35 years	74 (61.7%)	28 (23.3%)	18 (15.0%)	3.416	6	0.755
	35-45 years	37 (63.8%)	13 (22.4%)	8 (13.8%)			
	46-55 years	54 (60.0%)	24 (26.7%)	12 (13.3%)			
	> 55 years	63 (67.7%)	23 (24.7%)	7 (7.5%)			
**Religion**	Religious	159 (58.7%)	74 (27.3%)	69 (13.0%)	9.404	2	**0.009**
	Non-religious	69 (76.7%)	14 (15.6%)	592 (11.3%)			
**Place of** **residency**	Urban	211 (61.9%)	87 (25.5%)	43 (12.6%)	4.964	2	0.084
	Rural	17 (85.0%)	1 (5.0%)	2 (10.0%)			
**Place of** **workplace**	Urban	223 (62.6%)	88 (24.7%)	45 (12.6%)	2.958	2	0.228
	Rural	5 (100.0%)	0 (0.0%)	0 (0.0%)			
**Specialty**	Therapeutical	187 (64.3%)	71 (24.4%)	33 (11.3%)	1.814	2	0.404
	Surgical	41 (58.6%)	17 (24.3%)	12 (17.1%)			
**Professional** **experience**	< 10 years	83 (61.0%)	33 (24.3%)	20 (14.7%)	6.406	6	0.379
	11-20 years	47 (66.2%)	13 (18.3%)	11 (15.5%)			
	21-30 years	74 (100.0%)	22 (29.7%)	9 (12.2%)			
	> 30 years	55 (68.8%)	20 (25.0%)	5 (6.3%)			
**Experience in** **caring of patients with a terminal** **condition**	No experience	107 (64.5%)	35 (21.1%)	24 (14.5%)	4.054	6	0.669
	< 1 year	72 (63.7%)	28 (24.8%)	13 (11.5%)			
	1 year to 5 years	25 (55.6%)	15 (33.3%)	5 (11.1%)			
	> 5 years	24 (64.9%)	10 (27.0%)	3 (8.1%)			
**Experience in treating terminally ill**	Present	179 (67.8%)	61 (23.1%)	24 (9.1%)	12.983	2	**0.002**
	Non-present	49 (50.5%)	27 (27.8%)	21 (21.6%)			

Significant values are shown in bold.

The place of residence, workplace, specialty, professional experience, and experience in caring for patients with terminal conditions did not significantly influence attitudes towards the decision (p = 0.084, p = 0.228, p = 0.404, p = 0.379, and p = 0.669, respectively). However, experience in treating terminally ill patients significantly affected attitudes, with 67.8% (n=179) of those with experience supporting the decision compared to 50.5% (n=49) of those without experience (p = 0.002).

**Table 8** presents the attitudes of Lithuanian physicians towards a Living Will. Gender, age, place of residence, workplace, specialty, and experience in caring for or treating terminally ill patients did not significantly influence attitudes (p = 0.334, p = 0.180, p = 0.575, p = 0.097, p = 0.455, p = 0.118, and p = 0.881, respectively). Religiosity had a significant influence, with 66.1% (n=179) of religious respondents supporting the Living Will compared to 83.3% (n=75) of non-religious respondents (p = 0.007). Professional experience was also significant, with 78.7% (n=107) of respondents with less than 10 years of experience supporting the Living Will compared to 62.5% (n=50) of those with more than 30 years of experience (p = 0.036).

**Table 8:** The distribution of answers in the clinical case regarding Living-Will order according to different characteristics of respondents.

Demographic Variables		Number of Respondents			Significance		
		"Yes"	"No"	"Can't decide"	X²	df	*P*-value
**Gender**	Male	90 (70.3%)	27 (21.1%)	11 (8.6%)	2.191	2	0.334
	Female	164 (70.4%)	39 (16.7%)	30 (12.9%)			
**Age of** **respondents**	< 35 years	96 (80.0%)	15 (12.5%)	9 (7.5%)	8.884	6	0.180
	35-45 years	39 (67.2%)	11 (19.0%)	8 (13.8%)			
	46-55 years	59 (65.6%)	18 (20.0%)	13 (14.4%)			
	> 55 years	60 (64.5%)	22 (23.7%)	11 (11.8%)			
**Religion**	Religious	179 (66.1%)	58 (21.4%)	69 (13.0%)	10.007	2	**0.007**
	Non-religious	75 (83.3%)	8 (8.9%)	592 (11.3%)			
**Place of** **residency**	Urban	238 (69.8%)	64 (18.8%)	39 (11.4%)	1.108	2	0.575
	Rural	16 (80.0%)	2 (10.0%)	2 (10.0%)			
**Place of** **workplace**	Urban	251 (70.5%)	66 (18.5%)	39 (11.0%)	4.667	2	0.097
	Rural	3 (60.0%)	0 (0.0%)	2 (40.0%)			
**Specialty**	Therapeutical	203 (69.8%)	52 (17.9%)	36 (12.4%)	1.575	2	0.455
	Surgical	51 (72.9%)	14 (20.0%)	5 (7.1%)			
**Professional** **experience**	< 10 years	107 (78.7%)	19 (14.0%)	10 (7.4%)	13.484	6	**0.036**
	11-20 years	49 (69.0%)	9 (12.7%)	13 (18.3%)			
	21-30 years	74 (100.0%)	18 (24.3%)	8 (10.8%)			
	> 30 years	50 (62.5%)	20 (25.0%)	10 (12.5%)			
**Experience in caring of patients with a terminal condition**	No experience	127 (76.5%)	22 (13.3%)	17 (10.2%)	10.160	6	0.118
	< 1 year	76 (67.3%)	23 (20.4%)	14 (12.4%)			
	1 year to 5 years	30 (66.7%)	12 (26.7%)	3 (6.7%)			
	> 5 years	21 (56.8%)	9 (24.3%)	7 (18.9%)			
**Experience in treating terminally ill**	Present	186 (70.5%)	47 (17.8%)	31 (11.7%)	0.253	2	0.881
	Non-present	68 (70.1%)	19 (19.6%)	10 (10.3%)			

Significant values are shown in bold.

### 3.5 Logistic regression models

In this study, a logistic regression model was constructed to investigate the relationship between people’s perceptions of MAID and various socio-demographic factors. The primary objective was to better understand the factors influencing physician views on assisted suicide and euthanasia (both passive and active). Only factors that were significant in previous Chi-square tests and also had significant p-values in the regression model were included. Parameters that did not satisfy both criteria (such as the caring experience of less than 1 year in the first case scenario) were excluded from the model. All the models presented had an AIC value below 500, indicating a good fit.

The results of the binominal logistic regression models are shown in **Tables 9**–**14**. For example, the odds of acceptance in the first clinical case in the 35-45 age group are 57% lower than among respondents younger than 35 years, who serve as the reference group. The probability of acceptance of assisted suicide is 82.4% for respondents younger than 35 years (the reference group) and 67.2% for respondents aged 35-45 years. The difference in probabilities between these two groups is 15.2%. The largest reduction in the odds of acceptance, a decrease of 84%, was observed in the first case scenario among respondents aged 46-55 years.

**Table 9:** Binominal logistic regression model that predicts a prospective respondent’s attitudes toward assisted suicide depending on different circumstances.

Independent Variable	Reg. Coefficient β	Std. Error	Std. Deviation	Odds Ratio (95% CI)	P-value
Constant term	1.550	0.240	6.454	–	<0.05
Age:					
35-45 years	-0.831	0.368	-2.255	0.43 (0.21-0.90)	<0.05
46-55 years	-1.818	0.320	-5.668	0.16 (0.09-0.30)	<0.05
> 55 years	-1.658	0.317	-5.221	0.19 (0.12-0.35)	<0.05
Religious	-1.186	0.290	-4.085	0.31 (0.17-0.53)	<0.05
Work experience:					
11-20 years	-1.083	0.322	-3.360	0.33 (0.18-0.63)	<0.05
21-30 years	-1.450	0.316	-4.577	0.23 (0.12-0.43)	<0.05
> 30 years	-1.647	0.311	-5.288	0.19 (0.10-0.35)	<0.05
Caring experience:				
1-5 years	-0.825	0.343	-2.405	0.43 (0.22-0.86)	<0.05
> 5 years	-1.730	0.397	-4.349	0.18 (0.08-0.38)	<0.05

**Table 10:** Binominal logistic regression model that predicts a prospective respondent’s attitudes toward active euthanasia depending on different circumstances.

Independent Variable	Reg. Coefficient β	Std. Error	Std. Deviation	Odds Ratio (95% CI)	P-value
Constant term	1.285	0.221	5.800	–	<0.05
Age:					
35-45 years	-0.643	0.354	-1.817	0.43 (0.21-0.90)	<0.05
46-55 years	-1.418	0.306	-4.634	0.24 (0.13-0.44)	<0.05
> 55 years	-1.220	0.303	-4.021	0.30 (0.16-0.53)	<0.05
Religious	-0.853	0.274	-3.113	0.430 (0.24-0.72	<0.05
Work experience:					
11-20 years	-0.808	5.831	-2.567	0.45 (0.24-0.83)	<0.05
21-30 years	-1.178	0.308	-3.826	0.31 (0.17-0.56)	<0.05
> 30 years	-1.228	0.301	-4.075	0.30 (0.16-0.53)	<0.05
Caring experience:					
1-5 years	-0.825	0.343	-2.405	0.43 (0.22-0.86)	<0.05
> 5 years	-1.366	0.379	-3.604	0.25 (0.12-0.53)	<0.05

**Table 11:** Binominal logistic regression model that predicts a prospective respondent’s attitudes toward DNR order depending on different circumstances.

Independent Variable	Reg. Coefficient β	Std. Error	Std. Deviation	Odds Ratio (95% CI)	P-value
Constant term	4.077	0.712	5.719	–	<0.05
Religious	-2.287	1.026	-2.23	0.43 (0.24-0.72)	<0.05
Work experience:					
11-20 years	-0.808	5.831	-2.567	0.45 (0.24-0.83)	<0.05
21-30 years	-1.178	0.308	-3.826	0.31 (0.17-0.56)	<0.05
> 30 years	-1.228	0.301	-4.075	0.30 (0.16-0.53)	<0.05
Caring experience:					
1-5 years	-1.1673	0.535	-2.18	0.31 (0.11-0.92)	<0.05
> 5 years	-1.216	0.562	-2.163	0.30 (0.10-0.94)	<0.05

**Table 12:** Binominal logistic regression model that predicts a prospective respondent’s attitudes toward DNR without a patient’s consent depending on different circumstances.

Independent Variable	Reg. Coefficient β	Std. Error	Std. Deviation	Odds Ratio (95% CI)	P-value
Constant term	1.189	0.249	4.773	–	<0.05
Religious	-0.839	0.278	-3.018	0.43 (0.25-0.73)	<0.05
Treating experience	0.724	0.242	2.991	2.06 (1.28-3.32)	<0.05

**Table 13:** Binominal logistic regression model that predicts a prospective respondent’s attitudes toward Living-Will order depending on different circumstances.

Independent Variable	Reg. Coefficient β	Std. Error	Std. Deviation	Odds Ratio (95% CI)	P-value
Constant term	1.600	0.282	5.69	–	<0.05
Religious	-0.943	0.31	-3.039	0.39 (0.21-0.70)	<0.05
Work experience:					
21-30 years	-0.692	0.321	-2.156	0.50 (0.27-0.94)	<0.05
> 30 years	-0.794	0.311	-2.55	0.45 (0.24-0.83)	<0.05

**Table 14:** Binominal logistic regression model that predicts a prospective respondent’s attitudes toward assisted suicide due to mental illness depending on different circumstances.

Independent Variable	Reg. Coefficient β	Std. Error	Std. Deviation	Odds Ratio (95% CI)	P-value
Constant term	-1.011	0.206	-4.9	–	<0.05
Age:					
46-55 years	-1.185	0.407	-2.91	0.30 (0.13-0.65)	<0.05
Religious	-0.929	0.283	-3.275	0.39 (0.23-0.69)	<0.05
Work experience:					
21-30 years	-1.088	0.421	-2.581	0.27 (0.14-0.74)	<0.05

## 4 Discussion

### 4.1 Acceptance rates of MAID in case of somatic terminal condition

The inclusion strategies were designed to ensure a focused and relevant participant demographic that aligned with the study’s objectives. Distributing the survey both digitally through hospital intranets and physically in hospitals ensured the inclusion of older physicians, who may be less frequent internet users, thereby enhancing the diversity and representativeness of the participant sample. To further ensure that participants were exclusively Lithuanian physicians, several measures were implemented. The survey was conducted entirely in Lithuanian, with a clear description stating that it was aimed at assessing the attitudes of Lithuanian physicians. Additionally, the survey was distributed directly through hospital intranets in Lithuania. These targeted strategies effectively minimized the possibility of non-relevant respondents and ensured that the sample reflected the intended demographic—physicians practicing in Lithuania.

Our results brought information on the attitudes of physicians in Lithuania regarding various end-of-life decisions, such as assisted suicide, active euthanasia, non-resuscitation with and without consent, and the acceptance of living wills. The main finding indicates that in Lithuania, physicians’ attitudes towards end-of-life decisions vary based on factors such as religious beliefs, professional experience, and age. Notably, acceptance of assisted suicide for drug-resistant mental disorders is lower than for somatic disorders, but both are influenced by similar factors.

Based on our data, the acceptance rate of euthanasia and assisted suicide among physicians in Lithuania is approximately 61.5% in cases involving physical illness. This finding aligns closely with a study conducted in Israel, where 62% of physicians concurred that individuals should have the right to decide whether to expedite their own death (14). In Israel, the average attitude score towards passive/active euthanasia was 3.35 ± 0.79 out of 5 (15). In some countries, such as the USA, the acceptance rate is similar. For instance, 60% of physicians in the USA believe that Physician-Assisted Suicide (PAS) should be legal (16). In other countries, acceptance rates among physicians are higher; for example, in Finland, 69% of physicians fully or partly agreed that euthanasia should be accepted in cases of severe physical symptoms (17). Similarly, in India, 80% of physicians in a study expressed the opinion that euthanasia should be permitted for terminally ill patients (18).

In a broader context, these differences in acceptance rates may be partially influenced by each country’s cultural and historical background. The Lithuanian physicians tend to be quite conservative due to the lingering influence of the Soviet Union’s occupation and still dominating paternalistic principles in health care. However, Lithuania also has progressive elements, including a liberal parliament, open-minded citizens, and significant economic potential. The influence of Western ideology and a strong respect for individual freedom may also play a role in shaping the opinions of Lithuanian physicians regarding euthanasia and assisted suicide. This influence is even more evident when examining the acceptance rates among the general public in Lithuania, which are approximately 71% for both assisted suicide and euthanasia (13). Several reasons may explain why acceptance among physicians is lower compared to the general public. First, physicians often face a moral dilemma due to their professional standards, which obligate them to prioritize patient care and health. The responsibility of ending a patient’s life may conflict with these ethical obligations and instill fear of the associated responsibility. Additionally, while the general public may have a more abstract understanding of the process, physicians possess a deeper awareness of the complexities involved in determining a patient’s prognosis and the severity of their illness. This nuanced understanding can contribute to their more cautious stance on euthanasia and assisted suicide.

Such high acceptance rates among general public are more commonly seen in countries where MAID is legalized, such as Spain, where 84.5% of the population supports active voluntary euthanasia (19). In contrast, acceptance rates are significantly lower in other countries: 40% of the public in the United Kingdom (20) and 38.1% in Croatia (21).

### 4.2 Acceptance rates of MAID in case of drug-resistant mental illness

The situation changes significantly when discussing assisted suicide for drug-resistant mental illness. According to our study, only about 19.1% of physicians in Lithuania support this practice. The difference in acceptance rates between somatic and psychiatric disorders may be because people often do not perceive mental pain as being as real or severe as physical pain. This can be compared to the Netherlands, where euthanasia for psychiatric disorders is legalized. In the Netherlands, the percentage of physicians who considered performing euthanasia for patients with psychiatric disorders varied between 20% among medical specialists (which aligns with our data) and 47% among general practitioners (22). In Finland, just 12% of physicians fully or partially supported the idea that life becoming a burden is a valid justification for euthanasia, with an absence of a somatic illness (17). At the same time, most final-year nursing students supported the possibility of patients having access to euthanasia due to unbearable mental suffering (23). Again, public acceptance in Lithuania is slightly higher, with 40.2% of men and 30.5% of women in favor (13).

MAID for individuals with dementia as a comorbid condition, but not the primary illness, presents even greater challenges due to concerns about the patients’ competence in deciding to end their lives. 24% of Dutch general practitioners, 23% of clinical specialists, and 8% of nursing home physicians found it acceptable to perform euthanasia on patients with advanced dementia (24). The same study found that in the Netherlands, a total of 60% of the general public agreed that people with advanced dementia should be eligible for euthanasia.

Several factors may contribute to the differing perceptions of MAID practices in cases of somatic and mental disorders:

1. Mental health, by its nature, feels more familiar and accessible to people, as they experience it daily through changes in mood and emotional state. However, because people encounter drug-resistant mental illnesses far less often than somatic terminal diseases, they often lack a clear understanding of how severe and resistant these conditions can be. This leads to the belief that even drug-resistant mental illnesses might still respond to medication or other interventions. Unfortunately, this belief does not align with reality (25).
2. People often overlook the fact that, in both mental and somatic illnesses, the reasons for choosing to end one’s life prematurely are often the same. It is quite common to misunderstand the reasons behind end-of-life decisions for patients with a terminal somatic condition. They tend to assume that the primary driver is uncontrolled physical pain. However, data from Oregon (26) shows that physical pain does not even rank among the top five most common reasons for these decisions. Instead, the leading reasons include loss of dignity, autonomy, and control over bodily functions, as well as feeling like a burden to loved ones. This demonstrates that in both somatic and mental illnesses, the motivation for ending life is primarily psychological, not physical. However, this fact is often overlooked or misunderstood, whether due to lack of information or intentional avoidance, and this could explain the significant difference in acceptance of MAID in these two contexts.
3. The nature of mental disorders plays a significant role in how they are perceived. The lack of visual or laboratory diagnostic methods contributes to the stigmatization of these conditions and often leads to an underestimation of their severity by both the general public and the medical community.
4. Finally, in the case of mental illness, whether it is dementia or another condition, a more philosophical rather than scientific question arises: how do we define a person’s true will, and can a will influenced by a certain condition still be considered genuine? Perhaps the lower acceptance of assisted suicide for individuals with mental illnesses stems from concerns about whether they can fully understand and evaluate their own wishes.

The philosophical question about the true nature of a person’s will becomes especially significant, given that the ability to make informed decisions is often a crucial requirement when requesting to proceed any MAID practices (27). Some psychiatrists emphasize the complexity of assessing decision-making capacity for euthanasia in psychiatric patients, mentioning challenges like impaired judgment during depressive episodes and risks of external pressure. They advocate for stricter guidelines, including more consultations and extended evaluation periods, to ensure well-considered requests (28). It is also crucial to include psychiatric nurses in the discussion about euthanasia and unbearable mental suffering, as they are often the first to engage with patients expressing such requests. A study in Flemish psychiatric hospitals show that 53% of nurses had dealt with direct requests. While 84% did not object to euthanasia in psychiatric populations with unbearable mental suffering, 71% admitted lacking the knowledge and skills to address such requests effectively (29).

These findings highlight the need for comprehensive training, clear guidelines, and collaborative approaches involving both psychiatrists and nurses to ensure ethical and informed decision-making processes for patients seeking euthanasia due to unbearable mental suffering.

### 4.3 Acceptance rates of DNR and Living Will

In our study, the acceptance of Do Not Resuscitate (DNR) orders was the highest, with 92.2% agreeing when there is patient consent and 70.4% agreeing without it. In contrast, a smaller portion of physicians (63.1%) supported Living Will orders. This aligns with other studies, although in Israel, 40% of physicians have faced the dilemma of ordering a DNR (14). As we have shown earlier, religion significantly influences people’s attitudes towards MAID. Knowing that passive euthanasia is not considered a sin in many religious confessions, it is evident that DNR orders are viewed as more acceptable.

### 4.2 Prognostic factors for acceptance rates

#### 4.2.1 Gender

In our study, physicians’ gender did not influence their attitudes towards any MAID practices, unlike in the general Lithuanian population, where gender differences in attitudes toward euthanasia have been observed, as noted in previous research (13). This may show the role of equality developed through medical education and professional training in Lithuania, where both genders receive the same education and develop similar ethical perspectives, leading to a more uniform mindset regardless of gender.

This finding is consistent with data from Israel (15). There was also no influence of gender on attitudes towards MAID among nurses in Iran (30) and Polish medical students (31). However, a significant difference (p=0.0147) was observed among Indian physicians (18). Similarly, in Finland, gender was a significant factor, with females being more likely than males to object to euthanasia or physician-assisted suicide (17). Same is proven by data from Hong Kong- there was a higher acceptance of euthanasia among male medical students (32).

#### 4.2.2 Religion

Another significant factor associated with higher acceptance of euthanasia was being non-religious. This factor has been extensively studied in numerous previous studies. Religious individuals often view most practices of MAID as sinful, which prevents them from having a favorable attitude towards it. Lithuania is predominantly Catholic, with about 77.2% of the population identifying as Roman Catholic (33). The Catholic Church strongly opposes euthanasia, basing its stance on the Fifth Commandment, “Thou shalt not kill,” which it interprets as a prohibition against taking innocent life, including through assisted death. Pope Francis has also reaffirmed this view, calling euthanasia a “false solution” (34) that undermines human dignity. Consequently, many religious Lithuanians align with the Church’s position, contributing to widespread opposition to euthanasia in the country.

#### 4.2.3 Age

Age has proven to be a significant prognostic factor, with younger individuals supporting MAID more often. Several factors may explain this trend:

1. Influence of Soviet Past: Older generations were raised during the Soviet era (which lasted until 1991), a period marked by strict opposition to euthanasia. While the Soviet Union supported an official position of atheism and promoted antireligious policies, it also viewed euthanasia as morally unacceptable. Instead, it accepted the concept of “dysthanasia” (prolonging life through medical intervention regardless of quality of life). This cultural and ideological background likely influenced older individuals to have a more conservative view on end-of-life decisions.
2. Access to Information and Technology: Younger generations, in contrast, have grown up with greater access to technology and information. The ability to exchange ideas and learn about diverse perspectives has increased their appreciation for individual autonomy and the right to make personal decisions, including those about how to end one’s life. This exposure may contribute to their more progressive attitudes toward MAID practices.
3. Religiosity Among Older Generations: Despite growing up in the atheistic environment of the Soviet Union, older individuals in Lithuania tend to be more religious compared to younger generations. Since religious beliefs often oppose euthanasia, this factor may further reinforce their stricter views on MAID practices. The effect of religion has already been discussed.

However, this trend does not apply to DNR orders, as the acceptance of this practice is especially high across all age groups. Respondents of any age support it. There were also no significant differences in Living Will orders.

In India, there was a significant difference (p=0.0055) between physicians aged over 30 and those under 30 regarding the type of euthanasia they found justifiable (18). In Turkey, younger physicians (30-39 years) preferred the DNR option more than those aged 40-49 years (p<0.05) (35). Similarly, 5th-year medical students were 2.5 times more likely to believe euthanasia needs clear legal regulation compared to 2nd-year students (36). Same findings were observed among nursing students in Spain (37). Age is often a significant factor influencing attitudes toward euthanasia. Younger individuals in Lithuania and other countries tend to be more open-minded and have a greater respect for dignity. Additionally, new communication tools and social networking contribute to the liberalization of the younger population. However, it is important to note that some data contradicts this opinion. In the Netherlands, a factor associated with a positive attitude toward euthanasia was being between 40 and 69 years old, rather than younger.

#### 4.2.4 Experience in caring for terminally ill patients

Additionally, experience in caring for terminally ill patients has been proven to influence the general public in Lithuania in most cases (13). However, based on our study, this is slightly different for the physicians. Experience affects their opinions on euthanasia, assisted suicide, and DNR with patient consent, but not on other MAID practices. This may be because physicians tend to have stronger opinions, whether favorable or not, and factors such as experience in caring for terminally ill patients do not significantly affect their views. Different factors may explain why individuals with experience in caring for terminally ill patients tend to view MAID practices more negatively, but two key factors are likely the most significant:

1. People with experience in caring for terminally ill patients often have a deeper understanding of the main reasons why patients choose euthanasia. As highlighted earlier and supported by Oregon’s statistics, the primary motivations are psychological discomfort, such as loss of dignity or autonomy, rather than physical pain. Experienced caregivers recognize that these underlying issues can often be addressed, or at least partly controlled, through high-quality palliative care.
2. Experience in caring for terminal patients is often correlated with age, as older individuals tend to have more exposure to such cases over their life. This accumulated experience may shape a more categorical opinion on euthanasia, as older caregivers often hold more conservative views on the practice.

Some data suggest that attitudes toward MAID depend on the physician’s specialty, with oncologists showing higher acceptance (38). However, our data contradict this with a P-value= 0.579.

While our study primarily focuses on quantitative analysis, we recognize the importance of qualitative insights to better understand the reasoning behind physicians’ attitudes toward MAID. Previous qualitative research provides some valuable context. For instance, a Finnish study mentioned earlier revealed that physicians facing requests for assisted death often engage in detailed discussions about alternative care options, such as symptom management and palliative care (17). This fully supports our hypothesis that, with increasing experience, physicians come to understand that the most important factors for patients in terminal conditions are their mobility, independence, and mental well-being. Similarly, research from the Netherlands highlights that physicians experience significant emotional and ethical burdens when considering or performing euthanasia, balancing their duty to alleviate suffering with their responsibility to preserve life (22). These studies highlight the complex nature of such decisions, which are shaped by personal values, patient relationships, and professional responsibilities. Opinions on end-of-life decisions are influenced not only by factors like religion or experience, but also by individual values, emotional connections, and personal reflections. Such aspects cannot be fully understood through statistical analysis alone, emphasizing the importance of including qualitative data to capture physicians’ thoughts and doubts in these situations.

#### 4.2.5 Practical Implications

##### 4.2.5.1 Legislation

In Lithuania, proposals to legalize euthanasia have been introduced twice, yet neither came close to a parliamentary vote. This highlights the importance of reliable studies to understand the views of medical professionals, as physicians play a central role in these practices. Without such research, legislative proposals risk being disconnected from the realities of medical practice. Futile resuscitation is legally permitted in Lithuania as the only recognized end-of-life decision. In cases of extremely severe conditions, patients in critical care or their close relatives have the right to refuse resuscitation efforts (39).

##### 4.2.5.2 Education

Our findings also provide a clear foundation for improving medical education. By identifying patterns in physicians’ attitudes, such as generational differences or biases, this research can help design better training programs. These programs can focus on filling gaps in understanding and strengthening ethical decision-making, ensuring that physicians are better prepared to address complex end-of-life issues.

Studies have shown that simulation-based training positively influences the attitudes toward making end-of-life medical decisions. For instance, in a study from the United Kingdom, the average attitudes score toward end-of-life care among undergraduate nursing and medical students improved significantly from 119.6 to 128.4 after simulation training (40).Moreover, not only individual studies but also literature reviews show that palliative care simulations are highly effective in education (41).

##### 4.2.5.3 Palliative care

Furthermore, our findings, particularly from the block of questions attributed to drug-resistant mental illness and assisted suicide, suggest that physicians may undervalue psychological suffering compared to physical pain. The observed differences in attitudes toward assisted suicide in cases of mental illness versus physical illness lead us to hypothesize that this disparity might stem from a lack of understanding about the primary motivations for such decisions. Based on prior research, these motivations are often rooted in psychological distress, such as loss of dignity or autonomy, rather than uncontrolled physical pain. This highlights the need to prioritize education, financial support and policy efforts on improving palliative care approaches. Instead of focusing solely on symptom management through medication, more attention should be directed toward enhancing patients’ independence, mobility, and mental well-being to address the underlying causes of their distress. As mentioned earlier, implementing simulation-based training could also become a part of medical education in Lithuania, as palliative care is complex and focuses on improving the quality of life for patients and their families by addressing physical, emotional, social, and spiritual needs.

### 4.3 Conclusion

To summarize, our study highlights both commonalities with international trends and unique features reflecting Lithuania’s cultural and historical context. For example, gender did not influence physicians’ attitudes toward MAID, consistent with findings from Israel, Iran, and Poland, but contrasts with data from Finland and India. Religion remains a key factor, with Lithuania’s predominantly Catholic population aligning with the Church’s opposition to euthanasia.

Age also plays a role, with younger physicians generally more supportive of MAID, likely due to greater exposure to global perspectives and evolving views on autonomy, while older physicians’ conservative attitudes come from Soviet-era influences, religiosity, and extensive experience with terminal care. This experience often forms a preference for addressing psychological discomfort through palliative care rather than MAID. These findings have the potential to inform legislation, enhance medical education, and optimize financial allocation with a focus on palliative care.

### 4.4 Limitations of the study

Sample: The sample size for the study was determined using GPower and was adequate for most statistical analyses. However, a larger sample size is recommended to achieve smaller standard deviation windows and improve the precision of the results.

Demographic proportions: The unproportional representation of respondents based on some demographical data may affect the results. However, the overrepresentation of respondents working in urban settings is appropriate because most hospitals in Lithuania are located in urban areas due to the centralization of healthcare.

Data collection: the survey was conducted using both virtual and physical methods, which makes the potential for errors during the transcription of data from physical forms to Excel. To minimize this risk, a double-check procedure was implemented, with two researchers verifying the data to ensure accuracy.

Self-Selection: Participants who were less interested in the topic had the option not to complete the survey, which could potentially affect the results.

## Data Availability

The raw data supporting the conclusions of this article will be made available by the authors, without undue reservation.
